# An Audit of Patients with Dog-bite Wounds Presenting to a Tertiary Level Hospital Emergency Department in South Africa

**DOI:** 10.7759/cureus.6558

**Published:** 2020-01-04

**Authors:** Mzamo Nkomo, Zeyn Mahomed, Abdullah E Laher

**Affiliations:** 1 Emergency Medicine, Tambo Memorial Hospital, Johannesburg, ZAF; 2 Emergency Medicine, University of the Witwatersrand, Johannesburg, ZAF

**Keywords:** dog bites, dog bite wounds, rabies, wound irrigation

## Abstract

Background

Dog-bite wounds are a common emergency department (ED) presentation, accounting for approximately 5% of traumatic wounds in the US. However, only 20-50% of patients actually present to the ED for medical attention following a dog-bite wound.

Methods

This was a transverse, retrospective audit of medical records of patients that had presented to the Tembisa Provincial Academic Hospital ED with dog-bite wounds during the 2014 calendar year.

Results

Of the 269 patients that were included in the study, 148 (55%) were male. The median age of all study patients was 27 years (range: 3-77 years). Most patients presented between 18h00-24h00 (n = 111, 41.3%). Most wounds were sustained on the lower limbs (n = 80, 68.18%), followed by the upper limbs (n = 74, 28.03%). Patients who were ≤12 years of age had a higher prevalence of buttock/perineum (p = 0.0002) and head/face/neck (p = 0.009) wounds, whereas patients who were >12 years of age had a higher prevalence of lower limb wounds (p = 0.0006). Only 15 (5.6%) wounds were sutured, and antibiotics were prescribed to 120 (45.1%) patients. Tetanus toxoid vaccine (TTV) and the first dose of the rabies vaccine (RV) were administered to 152 (57.4%) and 240 (89.1%) patients, respectively.

Conclusion

Children are more likely to present with wounds to the head/face/neck or buttock/perineum regions, while adults are more likely to present with wounds to the lower limbs. Proper strategies should be implemented to ensure that clinicians adhere to the current antibiotics protocols as well as rabies and tetanus post-exposure prophylaxis (PEP)-prescribing guidelines.

## Introduction

Bite wounds are a common emergency department (ED) presentation accounting for approximately 5% of traumatic wounds and representing 0.3-1.1% of all ED presentations in the US [1,2]. However, only 20-50% of patients actually present to the ED for medical attention following dog bites in the US [3].

Despite a few controversies, there is consensus in the literature regarding the general management of dog-bite wounds. Specific treatment includes wound irrigation and debridement as well as tetanus and rabies post-exposure prophylaxis (PEP) as required [4]. Controversies relating to the management of dog-bite wounds include the routine prescription of prophylactic antibiotics and criteria for the suitability of primary wound closure [5].

Local data on the presentation and management of dog-bite wounds are limited [6,7]. Based on the estimated population size obtained from recent national census and extrapolation from data from the US, the incidence of dog-bite wounds in South Africa is estimated to be more than 200,000 cases per annum [8,9]. The management of patients with dog-bite wounds was shown to incur a huge expense at a tertiary level hospital located in the KwaZulu-Natal province of South Africa. The rabies immunoglobulin (RIG) was found to be the ninth most expensive drug purchased by the hospital [7]. In this study, we present the findings of a 1-year audit of patients that presented with dog-bite wounds to a single-center ED.

## Materials and methods

The research project was a transverse, retrospective audit of medical records of patients who had presented to the Tembisa Provincial Academic Hospital (TPAH) ED with dog-bite wounds during the 2014 calendar year (January 1-December 31). TPAH is an 880-bed institution situated north of Kempton Park in the Gauteng province of South Africa. It caters to an estimated population of 2.5 million people. Approximately 5,000 patients are treated in the ED monthly, a third of which are trauma-related cases. Permission to conduct the study and ethics approval was granted by the CEO of TPAH and the Human Research Ethics Committee (Medical) of the University of the Witwatersrand (certificate clearance no: M160943), respectively.

The researcher reviewed TPAH ED registers to identify patients that had presented with dog-bite wounds during the 2014 calendar year. In addition, surgical ward registers were also reviewed to identify patients that may not have been entered in the ED register. After selecting the relevant patient file numbers of potential study subjects, medical records were requested and obtained from the hospital records department. Relevant wards and doctors were also contacted in an attempt to locate medical records that were missing at the hospital records department. Medical records of patients that had presented for follow-up treatment of their dog-bite wounds after previously presenting to another medical facility and patients whose medical records could not be obtained or accessed were excluded from the study.

Relevant data were collected by the primary investigator who, prior to commencement of the study, had undergone informal training on the methods of data abstraction from medical records. The data collection process was monitored by the study supervisors. Collected data included sex, age, time of presentation, day of presentation, month of presentation, anatomical site/s of the wound, number of wounds, treatment administered (suturing of wounds, antibiotic prescription, tetanus PEP, rabies PEP), whether patients were admitted, duration of admission, and the presence of complications such as wound sepsis.

Conflicting data entries were resolved after consultation with the attending clinician or after mutual agreement between the primary investigator and the study supervisors. Inter-rater reliability was assessed by an independent researcher who was unaware of the study objectives and with experience in the methods of data collection from medical records. This was performed by re-abstracting data from a sample of 18 randomly selected medical records and comparing these to data collected by the primary investigator.

Data were analyzed using Stata version 13 (StataCorp Limited, College Station, TX). Categorical variables were presented using frequencies and percentages while continuous variables were presented using means and standard deviations. Association between the wound sites and patient age groups was assessed using Pearson’s Chi-squared test. The level of significance was set at α = 0.05. Reporting of the study findings adhered to STROBE (strengthening the reporting of observational studies in epidemiology) guidelines [10].

## Results

A total of the 294 patient names were identified from the ED registers, of which 27 were excluded. These included 14 patients whose medical records could not be found, four double entries, and seven patients in whom the diagnosis of a dog bite was incorrect. Subsequently, a total of 269 patients comprising 148 (55.0%) males and 121 (45.0%) females were included in the final sample. The median age of all study patients was 27 years (range: 3-77 years). The median age of female patients was 29 years [interquartile range (IQR): 21-38 years] was significantly higher than that of male patients (24 years, IQR: 11-34 years) (p = 0.0032). Most patients presented between 18h00 and 24h00 (n = 111, 41.3%), followed by 12h00 and 18h00 (n = 70, 26.0%), 06h00 and 12h00 (n = 64, 23.8%), and 00h00 and 06h00 (n = 24, 8.9%). There were no obvious trends regarding the day of the week or month of the year of the presentation. Details of the above are described in Table 1.

**Table 1:** Details of patients with dog bite wounds presenting to the emergency department

	N (%)
Sex	
Male	148 (55.0)
Female	121 (45.0)
Age group	
1–5	6 (2.2)
6–10	36 (13.4)
11–15	31 (11.6)
16–20	14 (5.2)
21–25	29 (10.8)
26–30	38 (14.1)
31-35	36 (13.4)
36–40	24 (8.9)
40–45	16 (5.9)
46–50	17 (6.3)
>50	22 (8.2)
Time of presentation	
00h00-06h00	24 (8.9)
06h00-12h00	64 (23.8)
12h00-18h00	70 (26.0)
18h00-24h00	111 (41.3)
Day of presentation	
Monday	41 (15.2)
Tuesday	37 (13.8)
Wednesday	37 (13.8)
Thursday	34 (12.7)
Friday	42 (15.6)
Saturday	37 (13.8)
Sunday	41 (15.2)
Month of presentation	
January	25 (9.3)
February	18 (6.7)
March	28 (10.4)
April	23 (8.6)
May	22 (8.2)
June	19 (7.1)
July	21 (7.8)
August	24 (8.9)
September	23 (8.6)
October	19 (7.1)
November	26 (9.7)
December	21 (7.8)

Most patients (n = 254, 95.1%) presented to the ED on the day of the bite itself. Overall, most dog-bite wounds were sustained on the lower limbs (n = 80, 68.18%) followed by the upper limbs (n =74, 28.03%). There were significant associations between the age group and the site of the dog-bite wounds. Patients ≤12 years of age had significantly higher rates of buttock/perineum (p = 0.0002) and head/ face/neck (p = 0.009) wounds, whereas patients >12 years of age had significantly higher rates of lower limb wounds (p = 0.0006). There were no significant age differences with respect to the upper limb (p = 0.154) or trunk (p = 0.092) injuries.

**Table 2:** Comparison of dog bite wounds at various anatomical sites between patients ≤12 years and patients >12 years of age The sum of percentages exceeds 100% as some patients had multiple wounds

	≤12 years (n = 67)	>12 years (n = 202)	P-value
Lower limb	34 (50.7%)	148 (73.2%)	0.0006
Upper limb	13 (19.4%)	57 (28.2%)	0.154
Trunk	10 (14.9%)	16 (7.9%)	0.092
Buttock/perineum	11 (16.4%)	5 (2.5%)	0.0002
Head/neck/face	8 (11.9%)	7 (3.4%)	0.009

**Figure 1:**
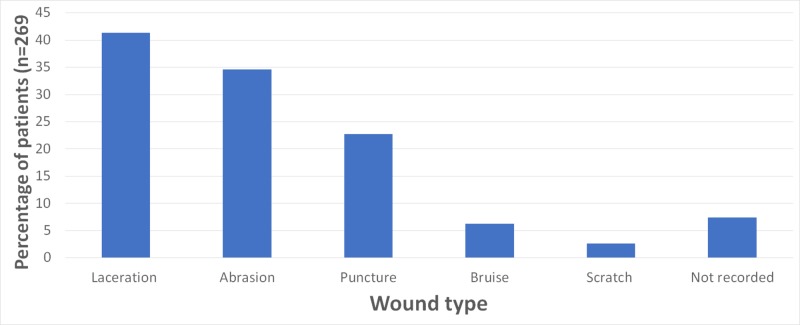
Types of dog-bite wounds The sum of percentages exceeds 100% as some patients had multiple wounds

Out of the 249 (92.57%) patients in whom the wound type was recorded, lacerations (n = 102, 40.96%) and abrasions (n = 85, 34.1%) were the most common wound types followed by puncture wounds (n = 56, 22.5%), bruises (n = 15, 6.0%), and scratches (n = 8, 3.2%) (Figure 1). Of the 195 (72.49%) patients in whom the number of wounds was recorded, the majority of the patients (n = 134, 68.72%) had sustained only one wound, while 39 (20.0%) patients sustained two wounds; 10 (5.1%) patients sustained three wounds, eight (4.1%) patients sustained four wounds, four (2.1%) patients sustained five wounds, and three (1.5%) patients sustained six or more wounds.

With regard to treatment, only 15 (5.6%) dog-bite wounds were sutured. Antibiotics were prescribed to 120 (45.1%) patients with cloxacillin (n = 71, 59.17%) being the most commonly prescribed antibiotic, followed by amoxicillin (n = 25, 20.8%), augmentin (n = 21, 17.5%), metronidazole (n = 11, 9.2%), gentamycin (n = 4, 3.3%), erythromycin (n = 3, 2.5%), ampicillin (n = 2, 1,7%), ciprofloxacin (n = 2, 1.7%), and penicillin (n = 2, 1.7%). Tetanus toxoid vaccine (TTV) was administered to 152 (57.4%) patients, while the first dose of the rabies vaccine (RV) was administered to 240 (89.1%) patients. However, only 173 (72.5%) patients completed the full course of RV. None of the patients received tetanus immunoglobulin (TIG), while only 3 (1.1%) patients received RIG. Only 22 (8.2%) patients required hospital admission. Of these, 14 (63.6%) patients were admitted for 3 days or less. Thirteen (4.9%) patients had presented to the ED with wound sepsis.

## Discussion

In this study, dog bites were slightly more common in males (55%) than females. This is in keeping with the results of other similar studies [3,6,11]. However, some researchers did not demonstrate a sex difference [12,13]. A possible reason for the higher incidence among males may be that males tend to be naturally more fearless than females and hence are likely to venture closer to an unknown or stray dog [14].

Dog bites were noted to be much less common in individuals younger than six years and older than 40 years with a greater decrease noted in individuals that were older than 50 years. A possible reason for this may be that the very young and the elderly tend to spend more time indoors and are therefore less likely to be exposed to stray and unfamiliar dogs. The overall median age for all patients in this study was 27 years, which is similar to the study conducted at the Ngwelezane Hospital in the KwaZulu-Natal province of South Africa in which the median age was 25 years [7]. In contrast, the median age was reported as 15 years in a study conducted in the US [3]; two Swiss studies among adult patients reported a median age of 36 years and 45 years, respectively [11,12]. In studies that included only children, the median age ranged from 5.7 to 7.4 years [6,13,15,16]. Children are more vulnerable to dog-bite attacks because of their small stature, and they are also more likely to incite a dog that is caring for its puppies or to play with a sick dog [17]. Various studies have reported that dog bites were more common in children younger than 10 years of age [3,7,15].

Dog bites can be expected to be more common after school and working hours. Indeed, most patients (41.3%) in this study presented to the ED between 18h00 to 24h00. Similar observations have been reported in the literature. In the audit that was conducted at the Red Cross Children’s Memorial Hospital in Cape Town, South Africa, 88% of children presented to the ED from 12h00 to 24h00 [6]. Similarly, in another Canadian audit, the median time of presentation for children who were victims of dog assaults was 17h30 [13].

No trend with regards to the day of the week was noted, which is in keeping with results from a study that was conducted in Austria [15] but is in contrast to the findings of two studies that were conducted in the US and Belgium, which reported a higher frequency of dog-bite wounds over weekends [3,16]. In this study, the percentage of dog-bite wounds varied minimally from month to month. There was no obvious evidence of a seasonal trend, which is in contrast to many studies that have demonstrated an increase in dog-bite wounds during the summer months when people and dogs spend more time outdoors [3,12,13,15,16]. A possible reason for this difference may relate to the fact that the South African winter is not as severe as that in other countries, making it more likely for both humans and dogs to be found outdoors in the South African winter.

In keeping with the literature [11,12], most dog-bite wounds in adults were located on the extremities. In contrast, children, likely due to their short body stature [13,15], had significantly higher rates of dog-bite wounds to the head/neck/face and buttock/perineum regions. In fact, some authors have noted that the location of dog-bite wounds is dependent on the age of the child. For example, pre-school children, who are more likely to be bitten at home by the family dog, tend to present with dog-bite wounds to the head/face/neck region, whereas, pre-teen children of school-going age, who are more likely to be bitten outdoor by an unfamiliar dog, tend to manifest dog-bite wounds to the buttocks, perineum, and lower limbs [6].

With regards to the management of dog-bite wounds, data pertaining to the specifics of wound debridement and irrigation were not reported in any of the hospital files. This is of concern, as these interventions are established as the most important steps to be taken in order to reduce the rates of wound infection [2,4] and are more important than the actual prescription of prophylactic antibiotics [18]. Despite evidence that dog-bite wounds can be safely sutured where suturing is not contraindicated [19,20], only 5.6% of dog-bite wounds in this study were sutured. This figure is low considering that the most common wound type was a laceration. In a national postal survey that was conducted in the UK, 60% of clinicians were opposed to suturing dog-bite wounds that were situated on body parts other than the face [21]. The results of this audit are in keeping with this misconception.

The routine use of prophylactic antibiotics is controversial. Current guidelines recommend that their use be restricted to dog-bite wounds deemed to be at high risk of infection [4]. However, the results of the published meta-analysis are contradictory with earlier studies demonstrating some clinical benefit [22]. The number needed to treat (NNT) to prevent one infection has been reported as 14 [23]. Recent results dispute earlier studies, especially when dog-bite wounds are appropriately debrided and irrigated [5,18]. Antibiotics were prescribed in 45% of dog-bite wounds in this study, the rationale for which was not clearly documented. It is unknown as to how many of those dog-bite wounds were indeed at high risk of infection.

In South Africa, 4 primary doses of the TTV are routinely administered to children between the ages of 6 weeks and 18 months, and a further 2 booster doses are administered at 6 years and 12 years of age [24]. Although the risk of acquiring tetanus is low after a dog-bite wound, it is recommended that a booster dose of the TTV is administered to the patient if he/she was last vaccinated more than five years ago when the wound is soiled and more than 10 years ago for clean wounds. Although the indications for tetanus PEP administration were not clearly documented, it was administered to 57.4% of individuals in this study. The number of previously immunized patients and the extent of wound contamination were not documented. It is also noted that TIG was not prescribed to any of the study patients. One possible explanation is that all patients were immunized and/or had received their booster vaccine within the recommended period. However, another plausible explanation is that clinicians may not be entirely familiar with current tetanus PEP guidelines.

With regards to rabies PEP, category 1 patients do not require any form of rabies PEP; category 2 patients do not require the RIG but require the RV, while category 3 patients require both the vaccine as well as the immunoglobulin [4]. Although rabies is a preventable disease, approximately 5-30 deaths per year are reported in South Africa, representing almost half of the global annual mortality. Half of the mortalities are reported in children less than 10 years of age. Various factors may account for the high mortality in South Africa. Firstly, many patients underestimate the severity of their injuries and often opt not to seek medical treatment. Almost 50% of laboratory-confirmed cases of rabies deaths in South Africa in 2008 had not presented to the ED. Secondly, some patients prefer to consult a traditional healer in certain parts of the country such as Kwa-Zulu Natal and Limpopo. Thirdly, studies have shown poor patient compliance with the vaccination schedule [4,25]. In an audit conducted in 2008 and 2009 in Kwa-Zulu Natal, 6% of the patients had defaulted on their rabies PEP [7].

In this study, an initial dose of RV was prescribed to 89.22% of patients. This figure suggests that 10.9% of dog-bite wounds were either risk category 1 (a lick of intact skin by a rabid dog), caused by an immunized dog, or the vaccine was not administered to all individuals that should have received it. On the other hand, even if there truly were 89.1% of rabies risk categories 2 and 3 dog-bite wounds, the full 4 or 5 dose course of the RV was prescribed to only 72.5% of patients. The administration of only one vaccine dose is unusual and not recommended. A minimum of two doses of RV is recommended for patients who had previously received rabies PEP [26,27]. Furthermore, RIG was prescribed to only 1.13% of study subjects. This figure seems very low as it is likely that there was more risk category-3 dog-bite wounds requiring RIG. In general, the risk category was not mentioned in the hospital records and therefore it is difficult to comment as to why a particular rabies PEP regimen was administered. A recent study illustrated how rabies PEP was inappropriately administered at a rural hospital in KwaZulu-Natal where rabies is endemic. RV was administered to 90%, 97%, and 99% of risk categories 1, 2, and 3 dog-bite wounds patients respectively, while RIG was administered to 53%, 84%, and 82% of categories 1, 2, and 3 patients respectively [7]. Hence, both RV and RIG were over as well as underprescribed. Several other studies demonstrated similar results. In Zaira, Nigeria, RV was not prescribed to 14.4% of deserving dog-bite- wound victims [28], whereas in a more recent Tanzanian study, 15% of probable rabies-exposed patients did not receive rabies PEP [29]. In yet another study based in KwaZulu-Natal, 17% of patients with grade 3 bites did not receive RIG [7]. A challenge is that rabies PEP is frequently out of stock or unavailable in hospital pharmacies. In a telephonic survey of health institutions in South Africa, 21% of facilities had no availability of either RV or RIG, 32% had only RV, 5% had only RIG, and 41% had availability of both [30].

Approximately 8% of study patients required admission for which the indications were not clear. This is similar to other studies where up to 96% of patients were discharged after sustaining a dog-bite wound [3,11]. However, studies in the pediatric population have reported admission rates of up to 15-27% [13]. This may be attributed to children requiring examination and management of their dog-bite wounds under anesthesia or that dog-bite wounds were more severe in children.

This study has some limitations. This was a retrospective single-center study with a relatively small sample size that had presented over a one-year period. Other limitations that are applicable to all studies of this nature (retrospective review of medical charts) include spurious findings and missing/conflicting data. However, these limitations do not affect the validity of our results. Hopefully, the findings of this study will pave the way for the public health impact of dog bite-related injuries to be better appreciated and targeted and will also encourage the implementation of appropriate preventative strategies going forward.

## Conclusions

Most patients with dog-bite wounds presented to the ED in the evening. We observed that children were more likely to present with dog-bite wounds to the head/face/neck or buttock/perineum areas and adults were more likely to present with dog-bite wounds to the lower limbs. We strongly recommend that appropriate strategies be implemented at hospitals and medical centers to ensure that clinicians adhere to the current antibiotic protocols as well as tetanus and rabies PEP-prescribing guidelines while attending to dog-bite wounds.
